# Analysis of complex chromosomal structural variants through optical genome mapping integrated with karyotyping

**DOI:** 10.3389/fgene.2025.1605461

**Published:** 2025-08-25

**Authors:** Xiaoxi Zhu, Huiling Zheng, Xue Wan, Hang Duan, Ying Qi, Weijia Tang, Fan Yang, Limei Yu

**Affiliations:** ^1^Key Laboratory of Cell Engineering of Guizhou Province, Guizhou Biomanufacturing Laboratory, Affiliated Hospital of Zunyi Medical University, Zunyi, China; ^2^Department of eugenic Genetics, Guiyang Maternity and Child Health Hospital, Guiyang, China; ^3^Research Center for Lin He Academician New Medicine, Institutes for Shanghai Pudong Decoding Life, Shanghai, China; ^4^Lishui Key Laboratory of Brain Health and Severe Brain Disorders, Lishui Second People’s Hospital, Lishui, China; ^5^Bio-X Institutes, Key Laboratory for the Genetics of Developmental and Neuropsychiatric Disorders (Ministry of Education), Shanghai Jiao Tong University, Shanghai, China; ^6^ Affiliated Hospital of Zunyi Medical University, Zunyi, China; ^7^Collaborative Innovation Center of Tissue Damage Repair and Regenerative Medicine of Ministry of Education, Zunyi Medical University, Zunyi, China

**Keywords:** recurrent spontaneous abortion, structural variations, chromosomal karyotype analysis, optical genome mapping, Kif7

## Abstract

**Background and Objective:**

Parental chromosomal structural variations (SVs) represent a primary genetic factor contributing to recurrent spontaneous abortion (RSA). Individuals carrying SVs with complex chromosomal rearrangements (CCRs) typically exhibit a normal phenotype but are at an increased risk of miscarriage. Current standard clinical detection methods are insufficient for the identification and interpretation of all SV types, particularly complex and occult SVs, thereby presenting a significant challenge for clinical genetic counseling. Leveraging the high-resolution capabilities of optical genome mapping (OGM) technology, this study aims to rapidly and accurately identify complex SVs in RSA couples. Furthermore, it seeks to conduct an in-depth analysis of the genetic information within the breakpoint regions, thereby providing a more comprehensive scientific foundation for genetic counseling of RSA couples at both the cellular and genetic levels.

**Material and Methods:**

This study involved the selection of nine subjects from two families who underwent genetic counseling at our hospital. Family 1 comprised a couple with the wife as a SVs carrier, and both her parents and brother were simultaneously analyzed for chromosomal karyotype. Family 2 included a couple with the husband as the SVs carrier, with his parents also undergoing chromosomal karyotype analysis. For SVs carriers whose karyotype analysis did not elucidate the recombination pattern, optical genome mapping (OGM) technology was utilized for further investigation, followed by Sanger sequencing to validate the OGM findings.

**Results:**

In Family 1, only the wife was identified as an SVs carrier. Initial chromosomal karyotype analysis suggested a karyotype of 46,XX,t (5; 6;8; 13; 15) (?). However, OGM analysis ultimately confirmed the karyotype as 46,XY,der (5)t (5; 13) (q35.2; q21.32), der (6)t (6; 8) (q25.3; q13.1)ins (6; 13) (q25.3; q21.32q21.33),der (8)t (6; 8) (q26; q13.1)ins (8; 13) (q13.1; q21.33q22.1),der (13)t (13; 15) (q21.32; q26.1)ins (13; 6) (q21.32; q25.3q26), der (15)t (5; 15) (q35.2; q26.1). Furthermore, OGM identified a novel translocation variant of the *KIF7* gene that is associated with recurrent miscarriage. In Family 2, both the husband and his maternal parent were identified as SVs carriers. Nuclear type analysis revealed a karyotype of 46,XY,?t (1; 6) (q42; p21) (husband) and 46,XX,?t (1; 2) (p31.1; q24.1),?t (1; 6) (q42; p21) (mother). Through OGM detection and analysis, the final karyotype was determined to be 46,XY,ins (1; 6) (q42.2; p22.3p11.3) (husband) and 46,XX,der (1)t (1; 2) (p31.1; q24.1)ins (1; 6) (q42.2; p22.3p11.3), der (2) t (1; 2), der (6)ins (1; 6) (mother).

**Conclusion:**

OGM technology facilitates the rapid and precise identification of complex chromosomal structural variations, effectively overcoming the limitations associated with traditional karyotype G-banding techniques in detecting intricate and cryptic SVs. This advancement substantially enhances the diagnostic rates of genetic etiology in patients experiencing RSA. The present study elucidates the specific manifestations of complex SVs using OGM technology, accurately pinpointing breakpoints and interpreting affected gene information. This provides novel reference approaches and evidence for disease assessment and genetic counseling in RSA patients. However, it is important to acknowledge certain limitations of this research: the study’s inclusion of only two RSA family cohorts (comprising nine participants) may limit the generalizability of its conclusions due to the small sample size, necessitating further validation through large-scale studies. Additionally, the causal relationship between *KIF7* gene dysfunction and recurrent miscarriage remains to be experimentally verified in subsequent research.

## 1 Introduction

Recurrent spontaneous abortion (RSA) significantly impacts the physical and mental health of women of childbearing age (He et al., 2024). While RSA is associated with various factors, including anatomical abnormalities, genetic predispositions, reproductive organ issues, endocrine dysfunctions, immune responses, and environmental influences (Andreescu et al., 2025), 50%–60% of RSA cases remain of unknown etiology (Di et al., 2023). The primary genetic cause of RSA is chromosomal abnormalities, this encompasses both embryonic chromosomal abnormalities and parental chromosomal structural variations (SVs). SVs are the main genetic contributors to early spontaneous abortion (Xue et al., 2023). Although carriers of SVs typically exhibit normal phenotypes, they have an increased risk of miscarriage due to unbalanced chromosomal rearrangements resulting from unequal crossover events during meiosis (Wright et al., 2025). Moreover, specific breakpoints in SVs carriers may disrupt haploinsufficient dosage-sensitive genes or their regulatory elements, increasing the risk for RSA and other clinical conditions (Zhou et al., 2023).

At present, diagnosis of such conditions primarily relies on chromosomal karyotype analysis, microarray technology, and high-throughput sequencing. However, these methods often fail to detect cryptic and complex SVs. For patients with unexplained RSA, enhancing diagnostic methods is crucial to providing more accurate genetic counseling and developing more effective fertility strategies.

Optical genome mapping (OGM) technology represents a new generation of high-resolution cytogenetic analysis capable of detecting SVs at the genome-wide level, including both chromosomal copy number and structural variations (Optical genome mapping to decipher the chromosomal aberrations in families seeking for preconception genetic counseling - Yin et al., 2025; Xie et al., 2024). OGM has been applied in the diagnosis of genetic disorders, particularly within the field of reproductive genetics (Xiao et al., 2024; Xie et al., 2024). In this study, we utilized OGM technology to identify SVs in two families with RSA cases and analyzed the gene information within the breakpoint regions. These findings could provide valuable insights for genetic counseling in clinical diagnosis and fertility planning at both the cellular and genetic levels.

## 2 Materials and methods

### 2.1 Subject information

This study involved the enrollment of two couples, each with reproductive health concerns and at least one member identified as a carrier of a SV, along with their family members, at our hospital. In total, the study included nine participants from two distinct families. In Family 1, both partners were 23 years old. Three years prior to the study, they experienced embryonic demise at 6 weeks of gestation, necessitating dilation and curettage. Additionally, 1 year before the study, they encountered an early pregnancy loss, prompting them to seek assisted reproductive treatment. Both partners exhibited normal phenotypes, denied any consanguineous relationships, and reported no exposure to teratogens, radiation, or substances such as nicotine, alcohol, or caffeine during pregnancy. There was no reported family history of genetic disorders, congenital malformations, or diabetes. Hysteroscopy of the female partner revealed a normal uterine cavity with endometrial hyperplasia and localized polypoid growth. Immunohistochemical analysis demonstrated DC138 positivity, with more than five plasma cells per high-power field (HPF). A vaginal color Doppler ultrasound indicated an endometrial thickness of 0.5 cm and identified a dominant follicle in the left ovary measuring 12 × 10 mm. Other biochemical and immunological evaluations were within normal ranges. The husband’s routine semen analysis revealed no apparent abnormalities (Supplementary Table S1). Following karyotype analysis, it was determined that the wife was a carrier of complex structural variants (SVs), leading to the inclusion of her parents and brother in the follow-up study (Supplementary Figure S1).

In Family 2, the husband (28 years old) and wife (26 years old) of the recurrent spontaneous abortion (RSA) couple visited the clinic twice due to miscarriages. The first miscarriage occurred at 7 months of gestation, with the patient reporting fetal abnormalities on ultrasound, although no documented records were available. This was followed by a spontaneous abortion at 2 months of gestation. Routine semen analysis indicated no significant abnormalities in the husband (Supplementary Table S2). Karyotype analysis confirmed that the husband was a carrier of SVs, which prompted subsequent genetic testing of his parents (Supplementary Figure S2).

Peripheral blood samples (5–10 mL) were collected from both subjects for relevant testing after obtaining written informed consent. The study protocol was approved by the Ethics Committee of the Affiliated Hospital of Zunyi Medical University (approval number KLL-2023-636). All methods were performed in accordance with the relevant guidelines.

### 2.2 Chromosome karyotype analysis

Whole blood (3–5 mL) was collected in a heparin anticoagulant tube (green tube). A volume of 0.8 mL of the collected blood was added to a peripheral lymphocyte culture medium and incubated at 37°C. After 68 h, 35 μL of 40 ng/mL colchicine were added to the culture vial using a 10 mL sterile syringe. The culture was mixed thoroughly and incubated at 37°C for an additional 3 h. Following incubation, the entire cell suspension was transferred to a 10 mL plastic centrifuge tube. Cells were then processed and stained using a cell harvester, a producer apparatus, and Giemsa dye. Chromosome karyotyping was performed using the Leica GSL-120 fully automated chromosome scanning system. Chromosome karyotypes were described according to the 2020 International System for Human Cytogenomic Nomenclature (ISCN 2020).

### 2.3 OGM analysis

Sample Collection: Human peripheral blood samples were collected using EDTA anticoagulant tubes and stored at −80°C (650 μL per sample, with at least two replicates per sample). Due to sensitivity to high temperatures, samples are recommended to be stored for no more than 5 days. Stability was assessed for up to 66 h at room temperature (22°C–25°C) and up to 6 h at elevated temperatures (30°C–40°C).

Genomic DNA Extraction and Marker Staining: Genomic DNA (gDNA) was extracted from the peripheral blood samples using the Bionano Prep SP-G2 Kit, following the manufacturer’s instructions for sample preparation. DNA concentrations were measured using the Qubit^®^ BR (Broad Range) dsDNA Assay Kit. A total of 750 ng of gDNA was extracted and labeled with the Bionano Prep DLS-G2 Kit. This labeling specifically targets the hexameric sequence motif (CTTAAG) within the genome, producing a green fluorescence signal, while the DNA backbone is stained blue, resulting in DNA molecules that display a blue sequence with green fluorescent signals.

Computer Detection and Data Analysis: The labeled DNA (recommended concentration: 4–16 ng/μL) was loaded onto a Saphyr chip for imaging and analysis. The DNA molecules were linearized using the chip’s specialized structures and then passed through nanochannels under the influence of electrophoresis. Images were captured to visualize the fluorescently labeled DNA. Post-imaging, data underwent quality control and whole-genome assembly using the Saphyr system’s software. The assembled data were then compared with a reference genome map for analysis.

### 2.4 Sanger sequencing

Based on the breakpoint location information obtained from the OGM results, primers were designed upstream and downstream of the breakpoint. PCR amplification was then performed, followed by agarose gel electrophoresis. The resulting bands were excised and purified, and first-generation sequencing was conducted. The sequence data were subsequently analyzed.

## 3 Results

### 3.1 Chromosome karyotype analysis

The pedigree demographic data and chromosomal karyotype results for two families are detailed in Table 1.

**TABLE 1:** Pedigree demographic data and chromosomal karyotype results.

	Family	Gender	Age (year)	Abortion (frequency)	Fertility (fetal)	Karyotype
Family 1	SVs carrier 1	Female	23	2	-	46,XX,t (5; 6;8; 13; 15) (?)
	Partner of SVs carrier 1	Male	23	2	-	46,XY
	Mother of SVs carrier 1	Female	48	-	2	46,XX
	Father of SVs carrier 1	Male	50	-	2	46,XY
	Brother of SVs carrier 1	Male	18	-	-	46,XY
Family 2	SVs carrier 2	Male	28	2	-	46,XY,?t (1; 6) (q42; p21)
	Partner of SVs carrier 2	Female	26	2	-	46,XX
	Mother of SVs carrier 2	Female	51	-	3	46,XX,?t (1; 2) (p31.1; q24.1), ?t (1; 6) (q42; p21)
	Father of SVs carrier 2	Male	53	-	3	46,XY

**FIGURE 1:**
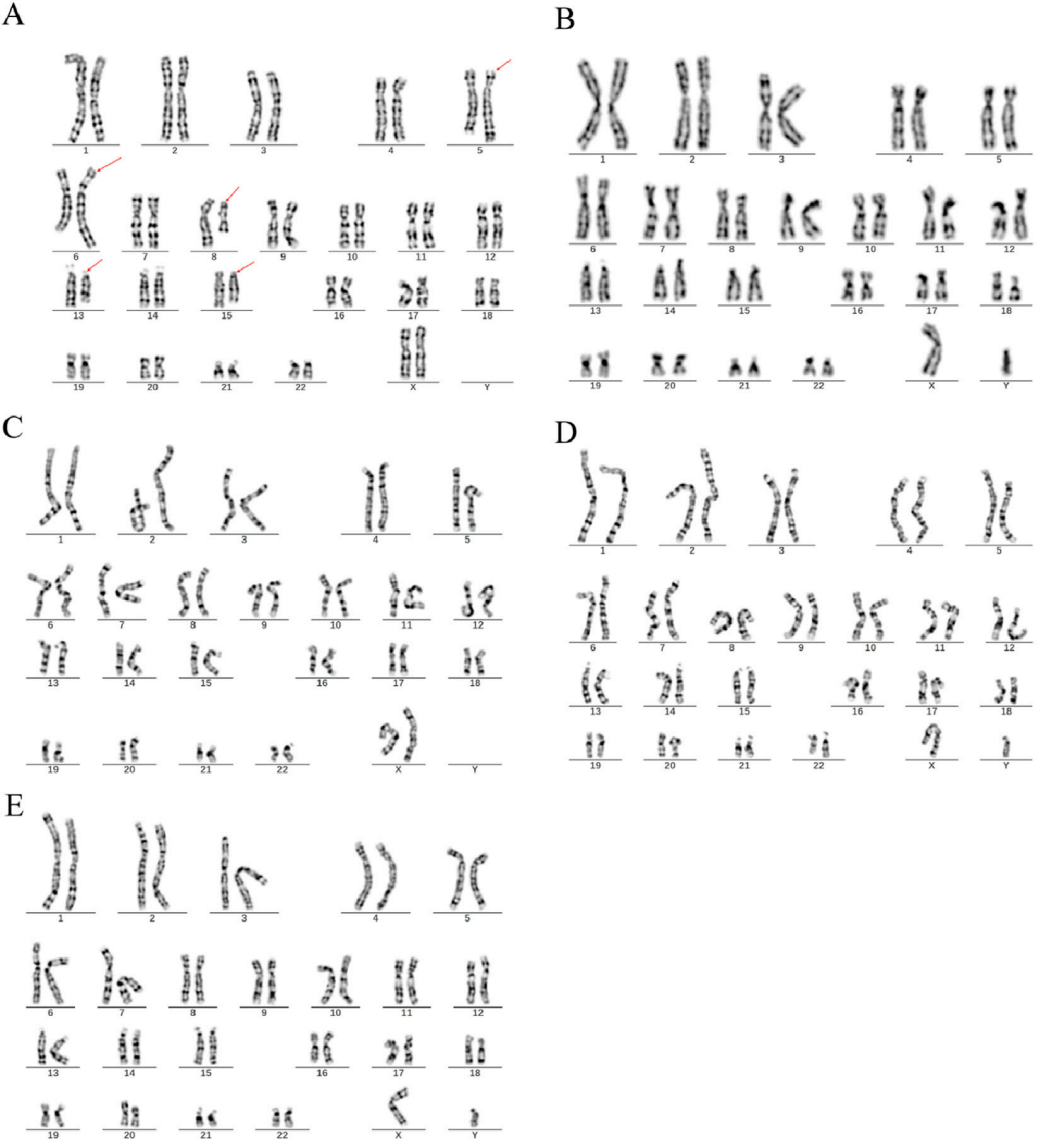
Chromosome karyotypes. **(A)** SVs carrier 1; **(B)** Partner of SVs carrier 1; **(C)** Mother of SVs carrier 1; **(D)** Father of SVs carrier 1; **(E)** Brother of SVs carrier 1.

In Family 1, the couple experiencing RSA underwent standard chromosomal karyotype analysis. The analysis revealed no abnormalities in the husband’s karyotype, whereas the wife was identified as a carrier of complex structural variations (SVs carrier 1). Due to the limitations in the technical resolution of this method, it was not possible to distinguish specific abnormal band information or potential chromosomal deletions or duplications. The karyotype of carrier 1 was preliminarily interpreted as a suspected balanced translocation, described as: 46,XX,t (5; 6;8; 13; 15) (?). No chromosomal abnormalities were detected in the karyotypes of Carrier 1’s parents or younger brother (Figure 1).

**FIGURE 2:**
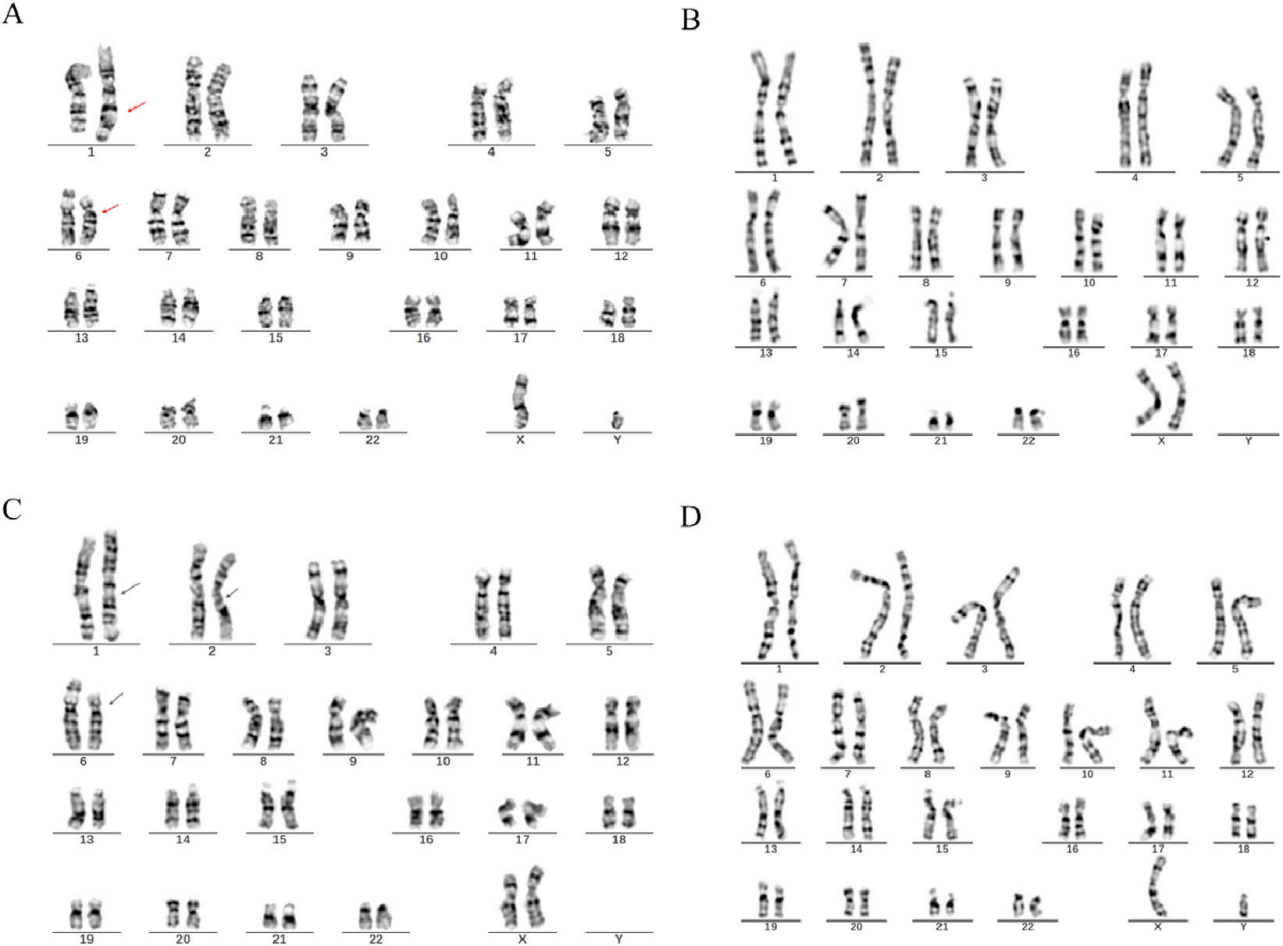
Chromosome karyotypes. **(A)** SVs Carrier 2; **(B)** Partner of SVs carrier 2; **(C)** Mother of SVs carrier 2; **(D)** Father of SVs carrier 2.

In Family 2, routine chromosomal karyotype analysis of the RSA couple indicated no abnormalities in the wife’s karyotype. However, structural abnormalities were identified in chromosomes 1 and 6 in the husband (SVs carrier 2), which were considered indicative of a suspected balanced translocation. The results were described as 46,XY,?t (1; 6) (q42; p21). The chromosomal karyotype analysis of the mother of Carrier 2 identified structural abnormalities in chromosomes 1, 2, and 6, which were suspected to be balanced translocations. The specific findings were 46,XX,?t (1; 2) (p31.1; q24.1) and ?t (1; 6) (q42; p21), with no abnormalities detected in the father’s chromosomes (Figure 2).

### 3.2 OGM analysis of SVs

**FIGURE 3:**
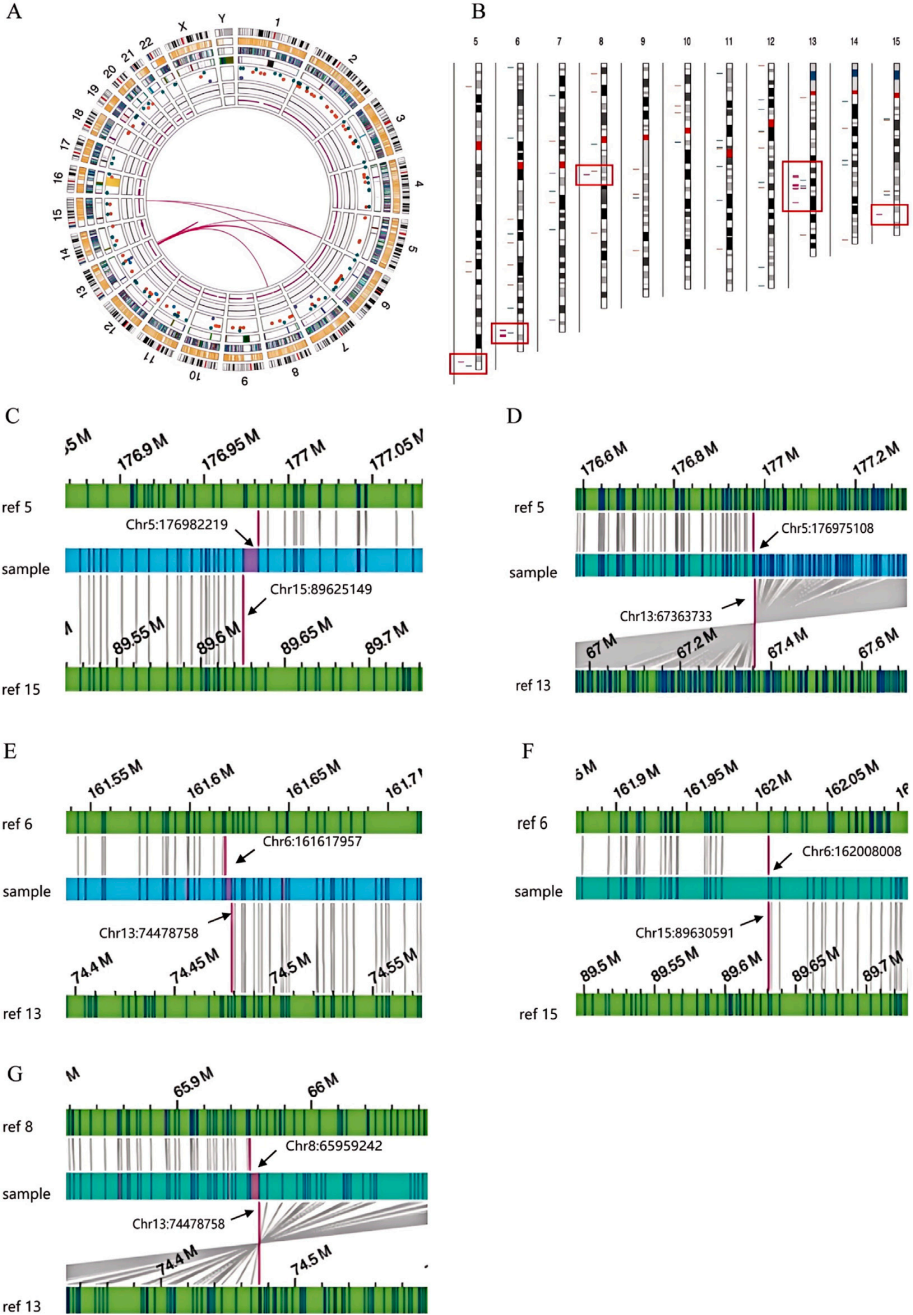
OGM circle plots and breakpoint plots for SVs carrier 1. **(A)** OGM circle plot; **(B)** Chromosome SVs distribution map (partial view); **(C)** Der (15) breakpoint plot; **(D)** Der (5) breakpoint plot; **(E,F)** Der (6) breakpoint plot; **(G)** Der (8) breakpoint plot.

To elucidate the breakpoints and recombination patterns of chromosomal SVs, OGM testing was performed on three SVs carriers. Both the test samples and the machine-processed data adhered to quality control standards. The analysis of OGM results indicated that SVs carrier 1 exhibited a complex rearrangement involving at least six chromosomes and nine breakpoints (Figure 3). Retrospective karyotype analysis determined the final chromosomal karyotype for this patient as 46,XY,der (5)t (5; 13) (q35.2; q21.32),der (6)t (6; 8) (q25.3; q13.1)ins (6; 13) (q25.3; q21.32q21.33),der (8)t (6; 8) (q26; q13.1)ins (8; 13) (q13.1; q21.33q22.1),der (13)t (13; 15) (q21.32; q26.1)ins (13; 6) (q21.32; q25.3q26), der (15)t (5; 15) (q35.2; q26.1).

**FIGURE 4:**
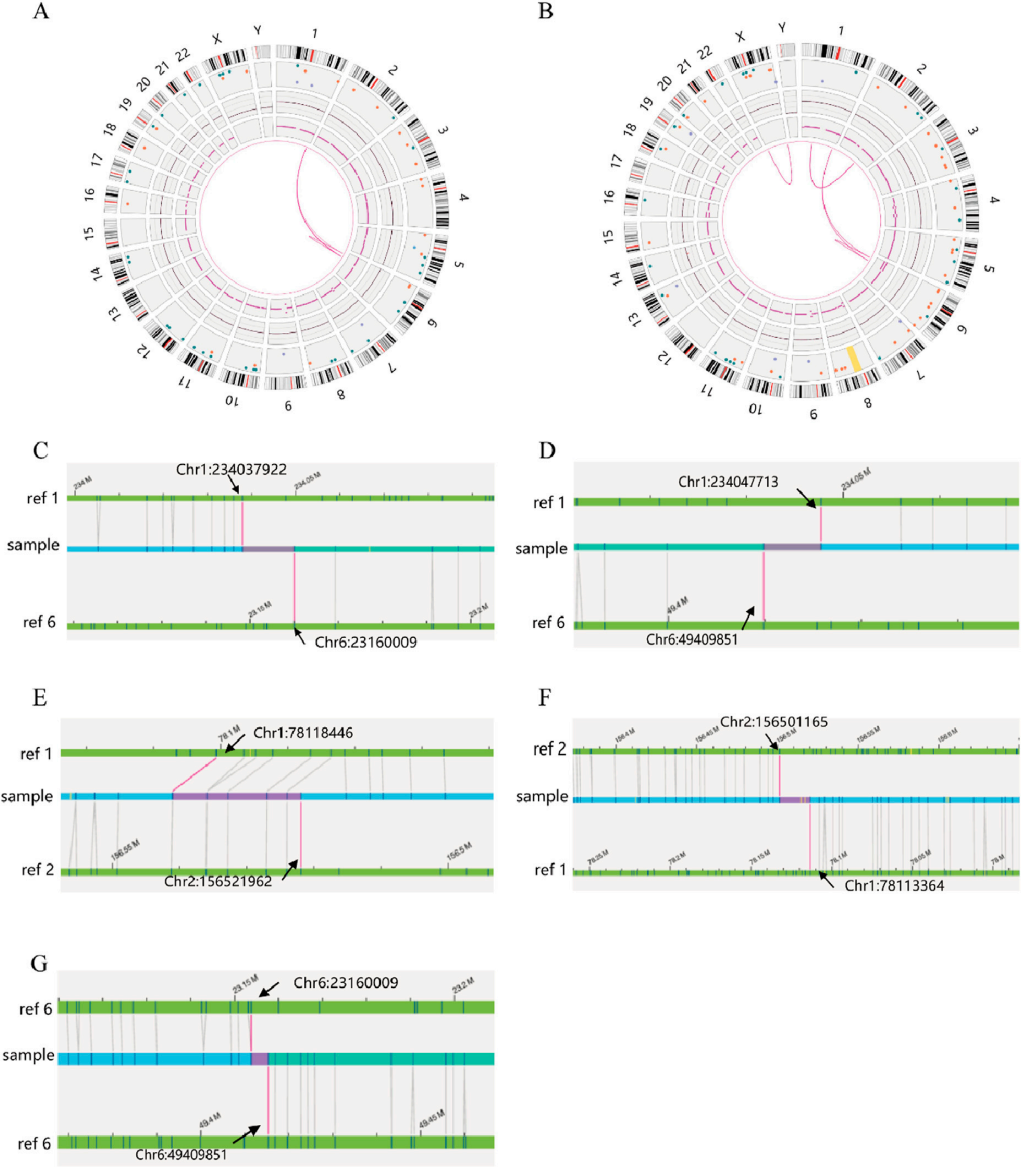
OGM circle plots and breakpoint plots for SVs carrier 2 and mother of SVs carrier 2. **(A)** OGM circle plot for SVs carrier 2; **(B)** OGM circle plot for mother of SVs carrier 2; **(C)** Der (1) breakpoint plots for SVs carrier 2; **(D)** Der (1) breakpoint plots for mother of SVs carrier 2; **(E)** Homologous Der (1) breakpoint plot for mother of SVs carrier 2; **(F)** Der (2) breakpoint plot for mother of SVs carrier 2; **(G)** Der (6) breakpoint plots for carrier 2 and mother of SVs carrier 2.

SVs carrier 2 was found to have two breakpoints on chromosome 6, with the broken fragments (approximately 26 Mb) inserted into chromosome 1 in a forward orientation. The final analysis for this case was determined to be 46,XY,ins (1; 6) (q42.2; p22.3p11.3). The mother of SVs carrier 2 was identified to have a complex rearrangement involving four chromosomes, including chromosomes 2 and X, with seven breakpoints. The comprehensive analysis revealed the following chromosomal rearrangements: 46,XX,der (1)t (1; 2) (p31.1; q24.1)ins (1; 6) (q42.2; p22.3 p11.3), der (2)t (1; 2), and der (6)ins (1; 6). Additionally, a minor recombination event was identified on the X chromosome itself (Figure 4).

### 3.3 Analysis of breakpoints and rearrangement structures of chromosomal SVs

**FIGURE 5:**
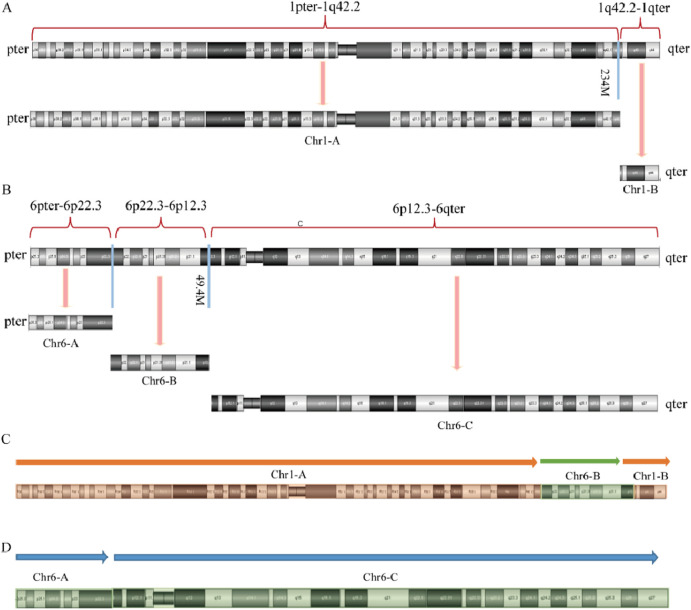
Diagram of abnormal chromosome SVs and derivative chromosome structures in SVs carrier 2. **(A)** Chromosome 1 SVs; **(B)** Chromosome 6 SVs; **(C)** Schematic diagram of the structure of derivative chromosome 1; **(D)** Schematic diagram of the structure of derivative chromosome 6.

Given that the SVs in carrier 2 are maternally inherited, elucidating the exact breakpoints and rearrangement patterns is crucial for providing informed genetic counseling to individuals with analogous complex chromosomal rearrangements. The structural anomalies present in chromosome 2 of SVs carrier 2 are depicted in Figure 5. Specifically, the structurally altered chromosome 1 exhibits a singular breakpoint, resulting in its division into segments Chr1-A and Chr1-B. Chromosome 6, on the other hand, displays two breakpoints, leading to the formation of three distinct segments: Chr6-A, Chr6-B, and Chr6-C. Notably, Chr6-B is interposed between Chr1-A and Chr1-B. Importantly, no potentially pathogenic genes associated with RSA were identified in proximity to these chromosomal breakpoints.

**FIGURE 6:**
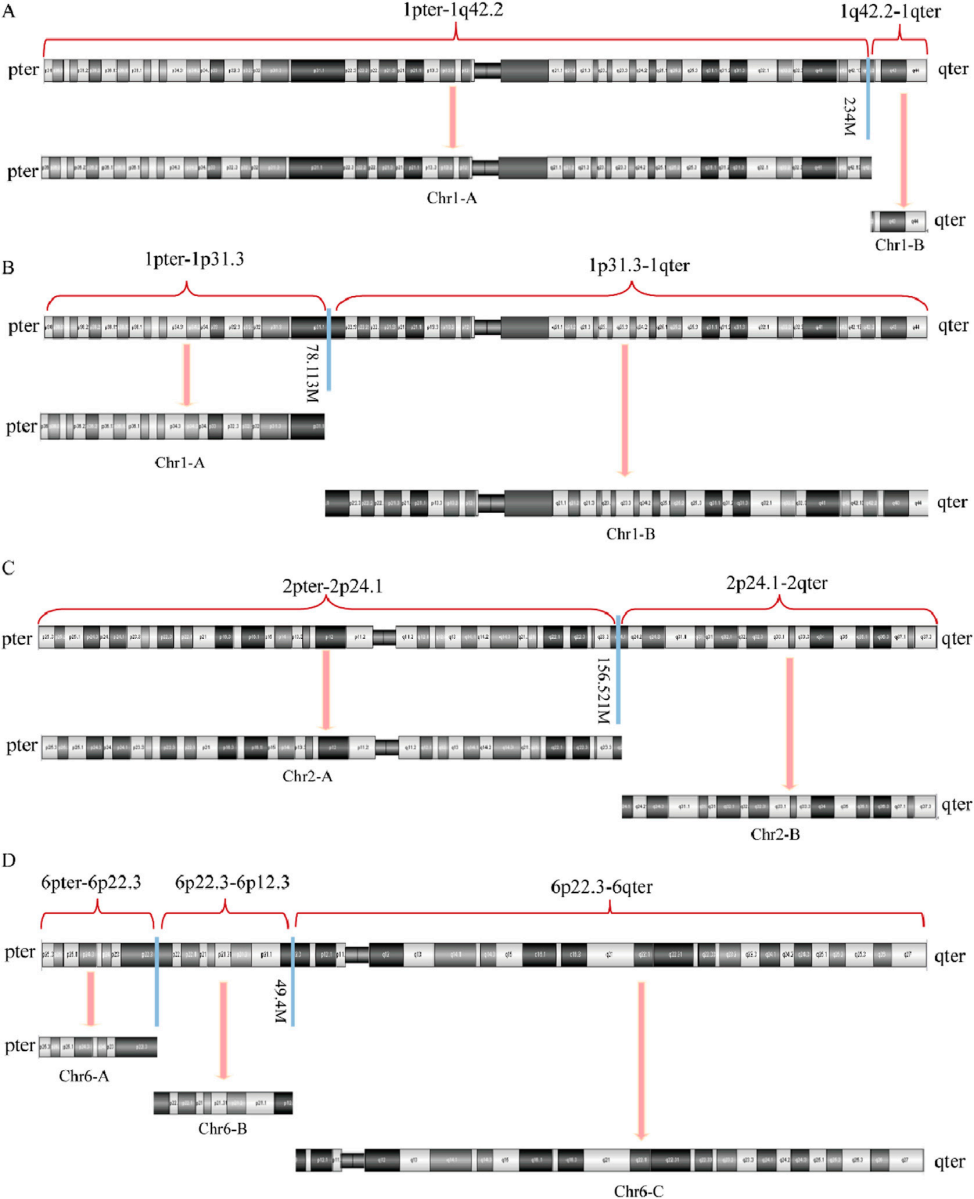
Diagram of abnormal chromosome SVs in mother of SVs carrier 2. **(A)** Chromosome 1 SVs; **(B)** Derivative chromosome 1 SVs; **(C)** Chromosome 2 SVs; **(D)** Chromosome 6 SVs.

**FIGURE 7:**
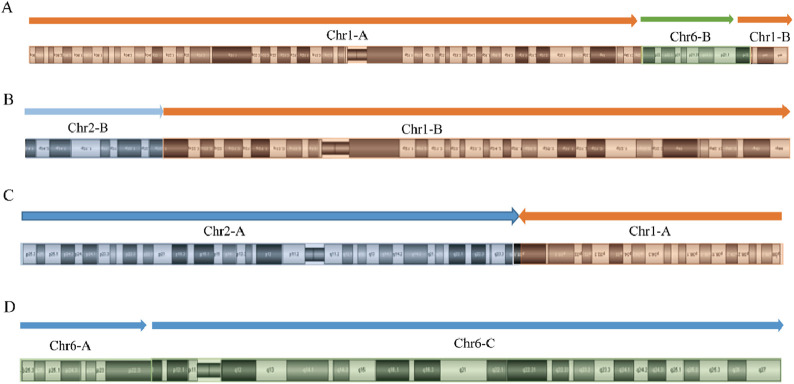
Diagram of derived chromosome structures in mother of SVs carrier 2. **(A)** Derivative chromosome 1 structure diagram; **(B)** Homologous derivative chromosome 1 structure diagram; **(C)** Derivative chromosome 2 structure diagram; **(D)** Derivative chromosome 6 structure diagram.

Figures 6, 7 present schematic representations of the structural chromosomal abnormalities, including breakpoints and rearrangements, observed in the mother of SVs carrier 2. The structural abnormalities observed on chromosomes 1 and 6 correspond to the breakage-rearrangement patterns identified in SVs carrier 2. However, additional balanced translocations were detected on another chromosome 1 and chromosome 2, suggesting that the chromosomal rearrangements in the mother of SVs carrier 2 are more intricate than those in SVs carrier 2 itself. Notably, no suspected pathogenic genes associated with RSA were identified near the breakpoints.

### 3.4 Validation of breakpoint locations by sanger sequencing

**FIGURE 8:**
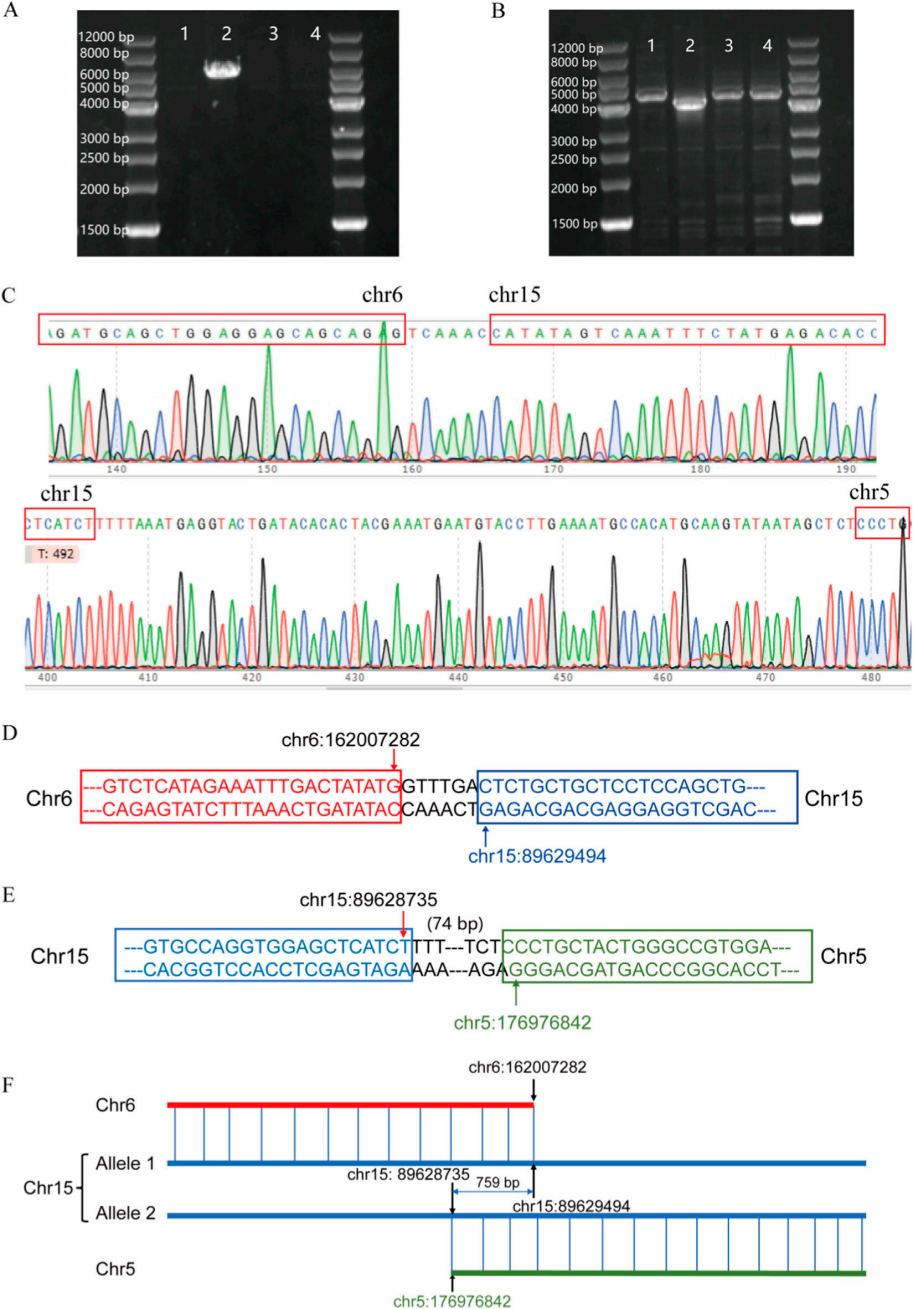
**(A)** t (6; 15) translocation PCR result; **(B)** t (5; 15) translocation PCR result; **(C)** Sanger sequencing verification results; **(D)** Schematic diagram of t (6; 15) translocation; **(E)** Schematic diagram of t (5; 15) translocation; **(F)** Schematic diagram of *KIF7* translocation.

OGM can rapidly and accurately detect complex chromosomal SVs, thereby increasing the diagnostic yield for the genetic causes of RSA. It can also identify balanced and cryptic SVs and provide information on genes located near breakpoint regions. Through PCR validation and Sanger sequencing, we confirmed that the inter-chromosome translocation breakpoints on chromosome 15 were located within the *KIF7* gene, which is suspected to be associated with recurrent miscarriage of patient 1. The *KIF7* gene is highly conserved and associated with an autosomal recessive embryonic lethal disorder known as fetal hydrolethalus syndrome. Sanger sequencing confirmed that the *KIF7* gene in this patient is disrupted due to translocations involving chromosomes 15, 5, and 6, leading to a complete loss of gene function. Both translocation variants identified are novel (Figure 8).

## 4 Discussion

Two or more clinical pregnancy losses are considered RSA (Tan et al., 2024). Chromosomal abnormalities are the primary genetic cause of RSA, with SVs such as balanced, Robertsonian, and inverted translocations being more prevalent than quantitative variations (Bozhinovski et al., 2024; Zhang et al., 2025). Balanced translocations are common chromosomal abnormalities, occurring in approximately 2.2% of RSA patients’ genomes. Accurate determination of translocation breakpoints is crucial for assisted reproductive technologies. Although balanced translocations do not involve the loss or duplication of genetic material, unequal chromosomal exchanges during meiosis can lead to RSA (Ren et al., 2023). Rarer types of aberrations, such as complex chromosomal rearrangements (CCRs) and balanced complex translocations (BCTs), are associated with an even higher risk of RSA (Liu et al., 2024; Wang et al., 2025).

A recent study reported a family with a balanced translocation t (5; 9) (p15; p24), whose members, despite being phenotypically normal, exhibit a significantly increased risk of spontaneous abortion and the birth of offspring with chromosomal abnormalities, such as Cri-du-chat syndrome (Zhao et al., 2025). In Family 1, recruited for this study, the wife was identified as a carrier of a rare complex chromosomal rearrangement involving multiple rearrangements among at least five chromosomes, specifically 5, 6, 8, 13 and 15. These variants can be categorized into two types: balanced and unbalanced translocations (Ardisia et al., 2024; Marković et al., 2025). Patients exhibiting unbalanced translocations are at a heightened risk of experiencing clinical issues, such as developmental delays (Zhou et al., 2025). Conversely, in cases of balanced translocations, even when the partner’s chromosomal composition is entirely normal, the probability of producing a balanced chromosomal zygote remains low. This significantly elevates the risk of recurrent spontaneous abortion or infertility (Ardisia et al., 2024; Vieira et al., 2024; Zhou et al., 2024). In Family 1, the wife’s OGM results indicated an absence of chromosomal duplications or deletions. Both her parents and younger brother exhibited normal karyotypes, suggesting she is a carrier of a complex chromosomal rearrangement. Despite this carrier status, she appears phenotypically normal, with no intellectual or developmental abnormalities. Nonetheless, the increased risk of recurrent miscarriage necessitates careful consideration. Routine genetic counseling for future pregnancies may involve preimplantation genetic testing for structural rearrangements (PGT-SR) or assisted reproduction through egg donation.

In family 2, the husband (SVs carrier 2) was found to be a carrier of structural variations in two chromosomes. His mother exhibited structural variations in three chromosomes, suggesting that the husband’s chromosomal variations were maternally inherited. During meiosis I, the mother’s three structurally abnormal chromosomes engage in a highly complex pairing configuration with six corresponding chromosomes, potentially leading to random segregation and recombination of these chromosomes, whether homologous or non-homologous. This process may result in the husband’s current karyotype. Traditional chromosomal karyotype analysis initially suggested that both SVs carrier 2 and his mother had ectopic chromosomes 1 and 6. However, subsequent Optical Genome Mapping (OGM) analysis revealed that both cases involved a 26 Mb fragment inserted into the long arm of chromosome 1. It is important to note that translocation and insertion represent distinct types of chromosomal rearrangements, each with unique mechanisms of gamete formation and associated genetic risks. Among these, insertion, excluding homologous translocation, poses the highest genetic risk. In alignment with our research findings, a study on rare chromosomal rearrangements involving six members from three-generation families has demonstrated that chromosomal insertions can lead to chromosomal instability in offspring, thereby increasing the risk of clinical manifestations such as intellectual disability, short stature, and congenital facial malformations (Nascimento et al., 2023). These studies underscore the benefits of OGM in clinical settings, particularly in addressing the limitations of traditional chromosomal detection technologies for identifying structural chromosomal variations. This advancement enhances the diagnostic rates of genetic etiologies for patients with RSA.

Traditional cytogenetic techniques, such as G-band karyotyping of peripheral blood lymphocytes, remain the standard diagnostic methods for detecting chromosomal rearrangements. However, the limited resolution of these techniques (>5–10 Mb), along with variability in sample preparation and laboratory quality control, results in the failure to detect many cryptic structural variations (Bao et al., 2025; Rao et al., 2023). In this study, karyotype analysis was effective in identifying carriers of chromosomal structural variations in families 1 and 2; however, it was unable to specify the type of variation, the precise location of the breakpoint, or the presence of deletions or duplications. Sequencing-based and array-based methodologies, such as copy number variation detection by sequencing (CNV-seq) and chromosome microarray analysis (CMA), improve the detection rate of chromosomal microdeletions and microduplications at the submicroscopic level. Nonetheless, these methods are inadequate for identifying balanced chromosomal translocations (Bao et al., 2025). Fluorescence *in situ* hybridization (FISH) is limited to the targeted verification of known structural variations, while whole genome sequencing (WGS) is suboptimal for structural variation detection due to the constraints of short read length technology in identifying repetitive regions (Budurlean et al., 2024; Meng et al., 2023).

OGM, a novel cytogenomic analysis technology, is capable of detecting all classes of structural variations and copy number variants with a resolution as fine as 500 base pairs. Furthermore, OGM can identify genes located within 10 kilobases of the breakpoint regions. Currently, this technology has been increasingly used in the study of genetic disorders, hematological diseases, and cancers (Ghabrial et al., 2024; Loghavi et al., 2024; Xiao et al., 2024). Numerous studies have demonstrated OGM’s capability to elucidate the relationships between genetic variants and clinical phenotypes based on the location of breakpoints (Dremsek et al., 2025; Lederbogen et al., 2024; Xiao et al., 2024).

In this study, we effectively utilized OGM to detect complex chromosomal rearrangements in SVs carriers, validate and refine chromosomal karyotyping results, identify specific variant types in each abnormal chromosome, and enhance the diagnostic yield for the genetic causes of RSA. Furthermore, OGM accurately identified the chromosomal breakpoint in the carrier from family 1 and discovered a novel translocation variant of the *KIF7* gene associated with miscarriage in this region, as verified by Sanger sequencing. This variant may be functionally compromised. Previous research has demonstrated that the *KIF7* gene is linked to fetal hydrops syndrome, an autosomal recessive genetic disorder (Putoux et al., 2011), and that *KIF*7 knockout in mice results in death either before or shortly after birth (Liem et al., 2009). Given that KIF7 is highly conserved, we speculate that the translocation variant involving the *KIF7* gene is likely the genetic cause of recurrent miscarriages in this patient. However, more precise conclusions would require integrating gene sequencing data from the patient’s partner. Unfortunately, due to specific constraints, we were unable to perform OGM testing and gene sequencing on the spouses of the RSA patients, limiting the amount of data that could be collected. To further validate the clinical relevance of *KIF7* variations in recurrent spontaneous abortion (RSA), we plan to investigate *KIF7* expression levels and related molecular pathological features in an expanded cohort of RSA patients and abortus tissues. Additionally, we intend to employ CRISPR-Cas9 gene editing to generate *KIF7* -knockout mice, providing an *in vivo* model to study the genetic and molecular mechanisms through which *KIF7* haploinsufficiency may lead to brain developmental abnormalities and miscarriage.

The findings of this study clearly illustrate the technical advantages of OGM. Nevertheless, it is important to acknowledge certain limitations inherent in the study: only two pairs of RSA families were included, comprising a total of nine participants. This limited sample size may impact the generalizability of the conclusions, necessitating further validation through large-scale studies. Furthermore, the study underscores certain limitations of the technology itself. Specifically, while the analysis of the OGM results for the mother of SVs carrier 2 identified the breakpoint and reconnection on chromosome 1 with high precision, OGM data alone was insufficient to conclusively determine whether a translocation or insertion involving chromosome 1 had occurred. This suggests the potential presence of an additional breakpoint and rearrangement, indicating that the abnormal SVs and derivative chromosomes may possess an alternative configuration (Supplementary Figure S3). OGM may produce similar outcomes under two distinct scenarios: (1) a break occurs in two homologous copies of chromosome 1, or (2) a break occurs in the long and short arms of the same chromosome 1, known as a cis configuration. The karyotype analysis suggests that the mother of SVs carrier 2 aligns with the second scenario. This case underscores the indispensable role of routine karyotype testing, highlighting that OGM does not serve as a “universal” diagnostic tool. The technology has limited resolution of highly repetitive or homologous complex genomic regions and is unable to detect small fragment variations (e.g., point mutations, microinsertions/deletions). In clinical practice, it is imperative to select appropriate testing methodologies tailored to the specific conditions of patients to facilitate auxiliary diagnosis.

This study, which combines chromosome karyotype analysis and OGM, has largely clarified two cases of familial chromosomal abnormalities. However, several challenges remain in clinical genetic counseling: 1). For SVs carrier 1: Conventional genetic counseling might suggest options such as PGT-SR or egg donation to achieve pregnancy. But should natural conception be completely ruled out? If natural pregnancy is considered, what are the chances of having a healthy offspring? 2). For SVs carrier 2: Why did the same chromosomal insertion lead to two consecutive abnormal pregnancies, while the mother, who has more complex chromosomal rearrangements, has three clinically normal children? Regarding question 1, using the mother of the SVs carrier 2 as a reference, even carriers of complex chromosomal SVs can have normal offspring. However, it is not possible to provide a simple numerical probability for a patient to have a healthy child through natural conception. Although it is theoretically possible to calculate the types of gametes produced, these numbers often lack practical significance, as they do not accurately reflect the real situation. The probability distribution of each potential gamete type is not equal, and the actual ratio of normal to abnormal gametes in carriers of structural chromosomal abnormalities is often much higher than the theoretical expectation. Concerning question 2, while more severe chromosomal abnormalities generally correlate with a lower ratio of normal to abnormal gametes, factors such as survivor bias or survival bias could explain the discrepancy in reproductive outcomes. Another possible reason is that the chromosomal rearrangements of the mother of the SVs carrier 2 are so complex that the genetic effects of deletions or duplications are severe enough to cause natural elimination of the fertilized egg early in pregnancy (biochemical pregnancy), which might go unnoticed as an early miscarriage. In contrast, SVs carrier 2 has a simpler chromosomal insertion involving a small segment, resulting in relatively minor genetic effects. This could explain why abnormal pregnancies in this patient progressed to two and 7 months, respectively. The two family pedigrees in this study demonstrate distinct chromosomal abnormalities, necessitating tailored genetic counseling approaches. The fundamental principle of genetic counseling lies in providing non-directive guidance rather than mandatory instructions. By fully utilizing available diagnostic technologies, we should deliver comprehensive and unbiased information to patients, thereby assisting them in making informed decisions while respecting their individual circumstances.

## 5 Conclusion

Our combined use of chromosomal karyotype analysis and OGM technology not only elucidated the mechanisms underlying chromosome rearrangements in RSA patients but also fully revealed the genetic causes of RSA by identifying genes involved at the breakpoints. This information is valuable for helping RSA patients select optimal reproductive strategies. The findings of this study also confirm that OGM can quickly and accurately detect complex chromosomal SVs, improve the diagnosis rate of genetic etiologies in RSA patients, and provide insights into both balanced and cryptic SVs. OGM addresses the limitations of current routine genetic diagnostics and offers additional guidance for clinical genetic counseling.

## Data Availability

The original contributions presented in the study are included in the article/Supplementary Material, further inquiries can be directed to the corresponding authors.
