# Supplementary material for: Analysis of complex chromosomal structural variants through optical genome mapping integrated with karyotyping
Source: Front Genet. 2025 Aug 25;16:1605461. doi: 10.3389/fgene.2025.1605461 (PMC12414755; doi:10.3389/fgene.2025.1605461)
Supplement: Supplementary file 1 [file Table1.docx]

Table 1. Pedigree Demographic Data and Chromosomal Karyotype Results.

| Family | | Gender | Age (year) | Abortion (frequency) | Fertility (fetal) | Karyotype |
| --- | --- | --- | --- | --- | --- | --- |
| Family 1 | SVs carrier 1 | Female | 23 | 2 | - | 46,XX,t(5;6;8;13;15)(?) |
|  | Partner of SVs carrier 1 | Male | 23 | 2 | - | 46,XY |
|  | Mother of SVs carrier 1 | Female | 48 | - | 2 | 46,XX |
|  | Father of SVs carrier 1 | Male | 50 | - | 2 | 46,XY |
|  | Brother of SVs carrier 1 | Male | 18 | - | - | 46,XY |
| Family 2 | SVs carrier 2 | Male | 28 | 2 | - | 46,XY,？t(1;6)(q42;p21) |
|  | Partner of SVs carrier 2 | Female | 26 | 2 | - | 46,XX |
|  | Mother of SVs carrier 2 | Female | 51 | - | 3 | 46,XX,？t(1;2)(p31.1;q24.1)，  ？t(1;6)(q42;p21) |
|  | Father of SVs carrier 2 | Male | 53 | - | 3 | 46,XY |
