# Supplementary material for: Analysis of complex chromosomal structural variants through optical genome mapping integrated with karyotyping
Source: Front Genet. 2025 Aug 25;16:1605461. doi: 10.3389/fgene.2025.1605461 (PMC12414755; doi:10.3389/fgene.2025.1605461)
Supplement: Supplementary file 2 [file DataSheet1.docx]

**Supplementary Data:**

**Table S1**. The sperm quality analysis report of partner of SVs carrier 1.

| Sperm classification | Number of sperm tested | Sperm concentration (10⁶/ml) | Total sperm count (10⁶) | Percentage  (%) |
| --- | --- | --- | --- | --- |
| PR (a + b class) (forward) | 1024 | 77.4 | 232.2 | 57.1 |
| NP (Class C) (non-forward) | 122 | 9.2 | 27.7 | 6.8 |
| IM (Class D) (non-active) | 648 | 49.0 | 147.0 | 36.1 |
| Total vitality (PR+NP) | 1146 | 86.6 | 259.8 | 63.9 |
| (PR+NP+IM) | 1794 | 135.6 | 406.9 | - |

**Figure S1**


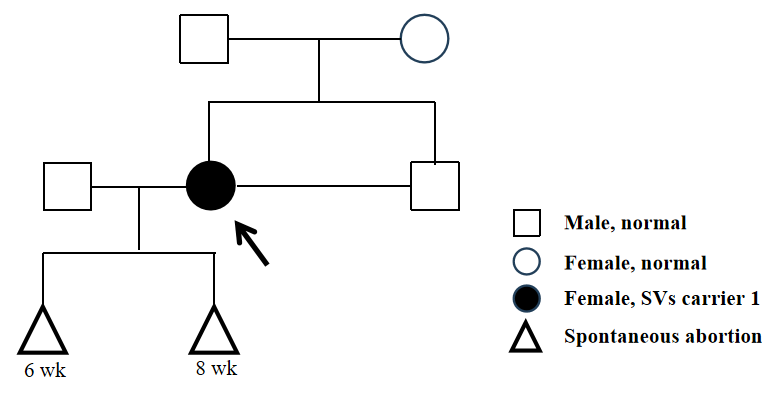


**Fig. S1：**The genealogy of family 1

**Table S2.** The sperm quality analysis report of SVs carrier 2.

| Sperm classification | Number of sperm tested | Sperm concentration (10⁶/ml) | Total sperm count (10⁶) | Percentage  (%) |
| --- | --- | --- | --- | --- |
| PR (a + b class) (forward) | 930 | 70.3 | 225.0 | 45.1 |
| NP (Class C) (non-forward) | 114 | 8.6 | 27.6 | 5.5 |
| IM (Class D) (non-active) | 1017 | 76.9 | 246.0 | 49.4 |
| Total vitality (PR+NP) | 1044 | 78.9 | 252.5 | 50.6 |
| (PR+NP+IM) | 2061 | 155.8 | 498.6 | - |

**Figure S2**


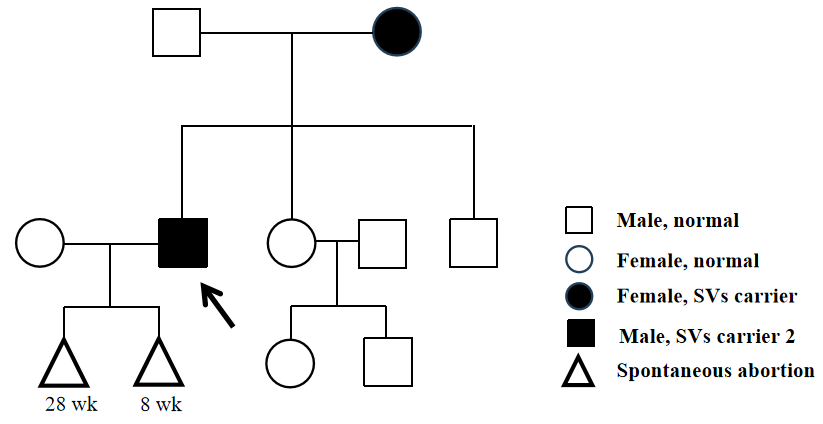


**Fig. S2：**The genealogy of family 2

**Figure S3**


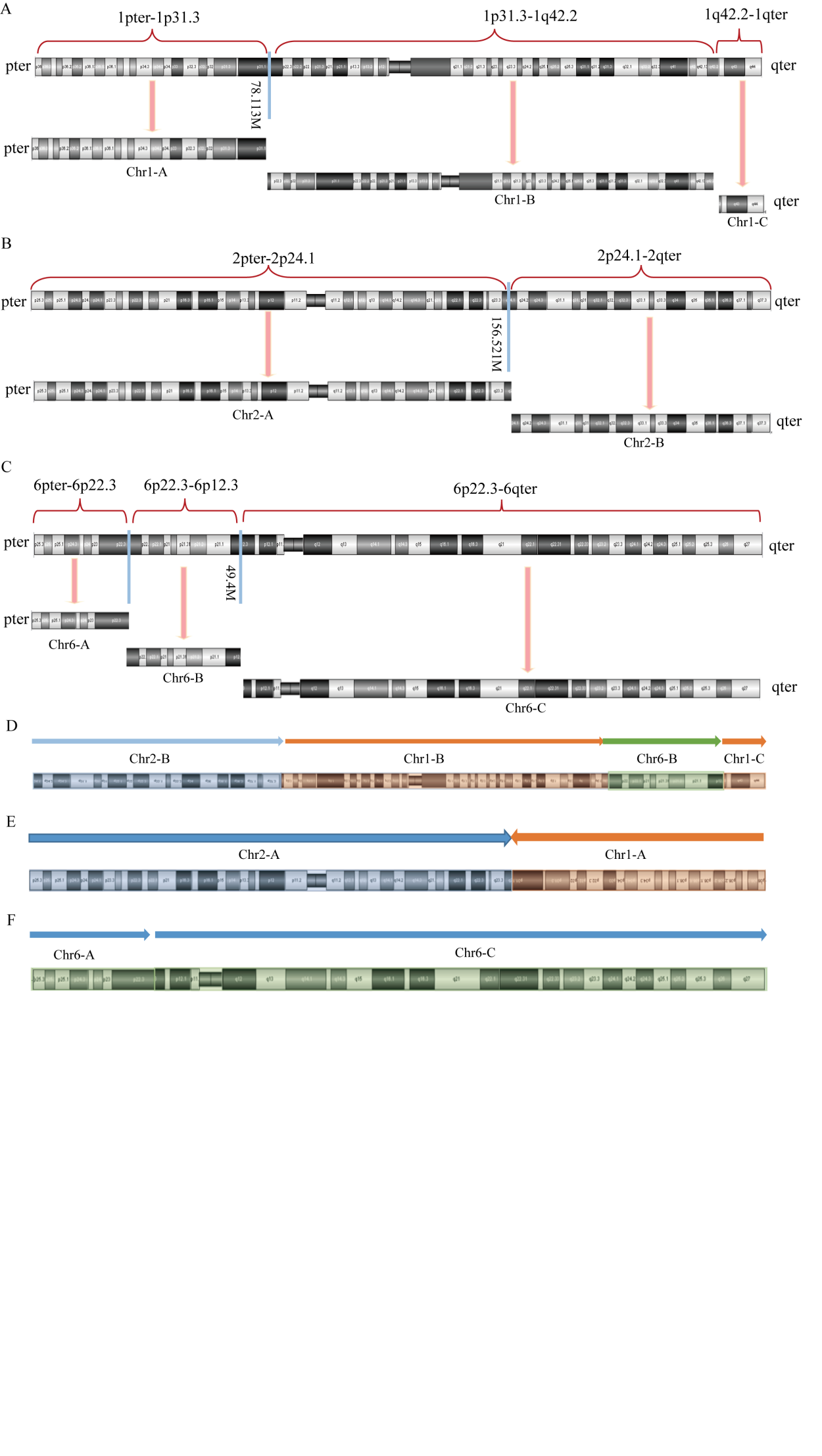


**Fig. S3：** Schematic Diagram of an Alternative Derivative Chromosome Structure in the Mother of SVs Carrier 2. A. Structural Variations (SVs) on Chromosome 1; B. Structural Variations (SVs) on Chromosome 2; C. Structural Variations (SVs) on Chromosome 6; D. Schematic Representation of the Structure of a Derivative Chromosome 1; E. Schematic Representation of the Structure of a Derivative Chromosome 2; F. Schematic Representation of the Structure of a Derivative Chromosome 6.
